# Emerging Cnidarian Models for the Study of Epithelial Polarity

**DOI:** 10.3389/fcell.2022.854373

**Published:** 2022-04-01

**Authors:** Lindsay I. Rathbun, Coralee A. Everett, Dan T. Bergstralh

**Affiliations:** Department of Biology, University of Rochester, Rochester, NY, United States

**Keywords:** cnidaria, epithelia, polarity, model organisms, apical-basal cell polarity

## Abstract

Epithelial tissues are vital to the function of most organs, providing critical functions such as secretion, protection, and absorption. Cells within an epithelial layer must coordinate to create functionally distinct apical, lateral, and basal surfaces in order to maintain proper organ function and organism viability. This is accomplished through the careful targeting of polarity factors to their respective locations within the cell, as well as the strategic placement of post-mitotic cells within the epithelium during tissue morphogenesis. The process of establishing and maintaining epithelial tissue integrity is conserved across many species, as important polarity factors and spindle orientation mechanisms can be found in many phyla. However, most of the information gathered about these processes and players has been investigated in bilaterian organisms such as *C. elegans, Drosophila*, and vertebrate species. This review discusses the advances made in the field of epithelial polarity establishment from more basal organisms, and the advantages to utilizing these simpler models. An increasing number of cnidarian model organisms have been sequenced in recent years, such as *Hydra vulgaris* and *Nematostella vectensis*. It is now feasible to investigate how polarity is established and maintained in basal organisms to gain an understanding of the most basal requirements for epithelial tissue morphogenesis.

## Introduction

Cell polarity defines specific spatial and functional domains within a cell through the asymmetric positioning of cellular components such as proteins, organelles, and cytoskeletal components. Epithelia are polarized tissues that perform specialized functions, typically at the boundary between an organ and the external environment. Cell polarity establishment and maintenance is vital for these functions; directional processes such as secretion, nutrient uptake, and signaling require a defined apical and basal surface to occur successfully (Humbert et al., 2008; St Johnston and Ahringer 2010). The importance of epithelial polarity is also highlighted by the observation that its loss is a common feature of malignancy (Bergstralh and St Johnston 2012; Williams et al., 2017; Jung et al., 2019; Tenvooren et al., 2019; Catterall, Lelarge, and McCaffrey 2020; Che et al., 2021; Tilston-Lunel et al., 2021).

**FIGURE 1:**
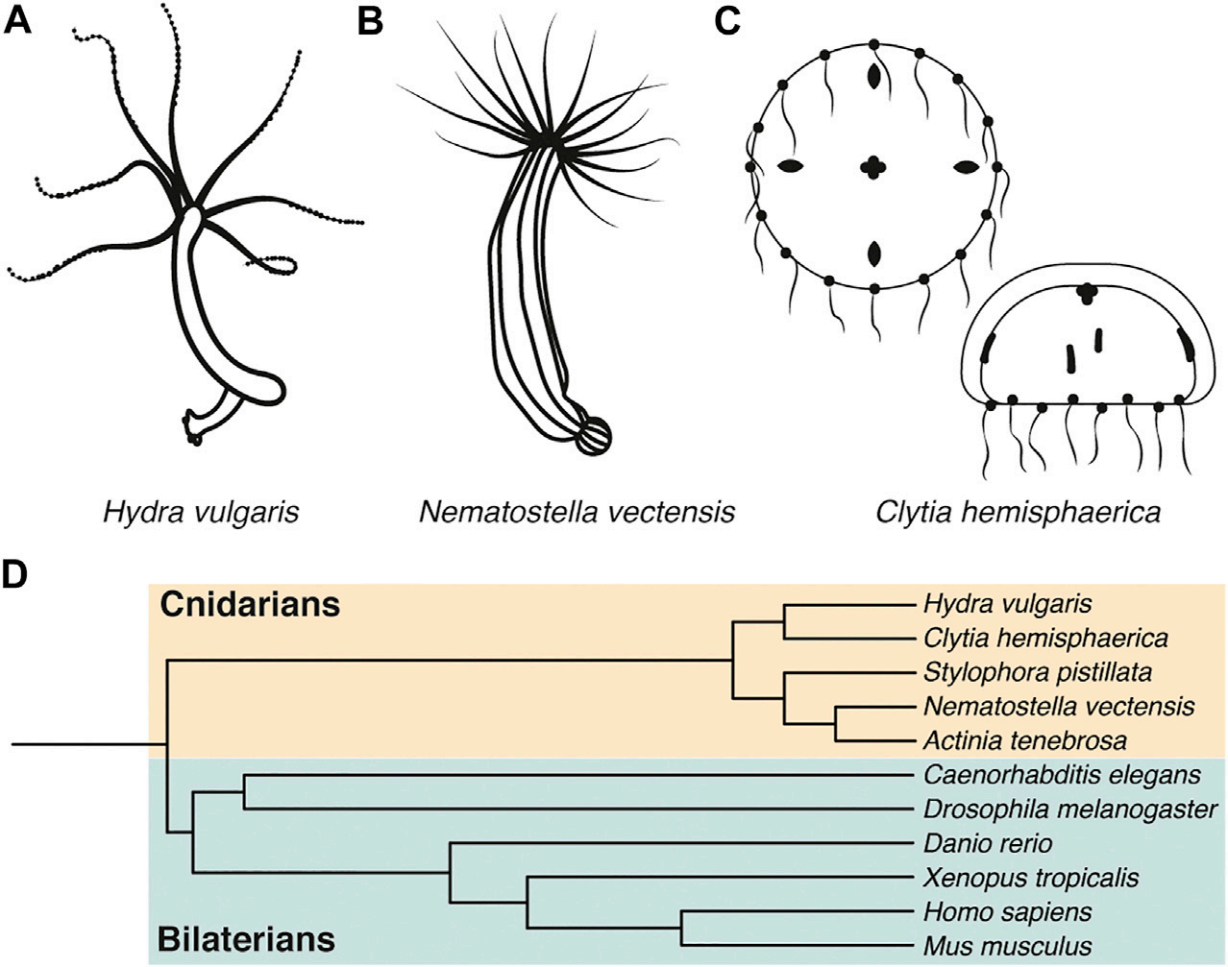
*H. vulgaris*, *N. vectensis*, and *C. hemisphaerica* are emerging model systems for polarity studies. **(A-C)** Representative drawings of *Hydra vulgaris*, *Nematostella vectensis*, and *Clytia hemisphaerica*. **(D)** Evolutionary tree depicting cnidarian and bilaterian model organisms.

For decades, studies of epithelialization and polarity establishment have focused largely on well-established bilaterian models, namely *C. elegans*, *Drosophila*, and mammalian systems. As with all biological model systems, however, each of these organisms comes with its own set of technical and genetic caveats. In this brief article we highlight the utility of cnidarian animals to study the process of epithelial polarity establishment and maintenance (Figure 1). We argue that these animals represent a promising yet under-utilized class of model organisms. Cnidaria and Bilateria are both phyla under the larger Eumetazoan subkingdom. Consistent with previous work, we show here that cnidarians share polarity establishment factors with bilaterians, within a simpler body plan. They also possess regenerative capabilities and a robust ability to reorganize upon dissociation, furthering their potential to push the field of polarity establishment and maintenance forward.

### Advantages to Phylogenetically Basal Model Organisms and Examples

There are some significant technical advantages to cnidarian models, an example of basal metazoans that diverged from bilaterians millions of years ago. Firstly, these simple organisms have robust regeneration capabilities that can be harnessed for use in studying polarity and cell sorting mechanisms (Seybold, Salvenmoser, and Hobmayer 2016; Cochet-Escartin et al., 2017; Skokan, Vale, and McKinley 2020). *Hydra vulgaris* and other cnidarians can completely regenerate from dissected tissue over the span of several days (Tucker and Adams 2014). Cnidarians are also able to reassemble from a completely dissociated cell suspension after mechanical or enzymatic dissociation (Tucker and Adams 2014; Cochet-Escartin et al., 2017). This provides an opportunity to follow the process of polarity establishment from a dissociated group of cells to a functional multicellular tissue in an *in vivo* animal context. This has previously been accomplished in polarized cell culture models such as MDCK cysts (Rodriguez-Boulan, Kreitzer, and Musch 2005; Martin-Belmonte et al., 2007; Mellman and Nelson 2008; Bryant et al., 2010) and three-dimensional organoid culture systems (Li et al., 2018; Serra et al., 2019; Lukonin et al., 2020; Rosenbluth et al., 2020; Schuster et al., 2020; Hendriks et al., 2021), however these models would not develop into a fully functional organism like an animal model such as *Hydra vulgaris*.

Cnidarian model systems can also be tailored to investigate how polarity establishment mechanisms differ between tissues. In addition to total dissociation protocols, there are established techniques to isolate particular tissues for site-specific studies. For example, *Hydra* mesoglea has been isolated through a detergent extraction and freezing protocol in order to investigate the extracellular matrix proteins within (Tucker and Adams 2014). Since extracellular matrix proteins can influence polarity establishment (reviewed in (Manninen 2015)), this protocol could be used to determine how extracellular matrix influences polarity establishment in *Hydra*, and possibly modified to investigate additional structures within the *Hydra*. Additionally, primary cell cultures can be created from cnidarian tissues for more in-depth studies, such as those generated from the cnidarian *Anemonia viridis* for use in tissue-specific and pluripotency marker expression studies (Ventura et al., 2018), providing another manner in which to study polarity establishment in specific cnidarian tissues.

**FIGURE 2:**
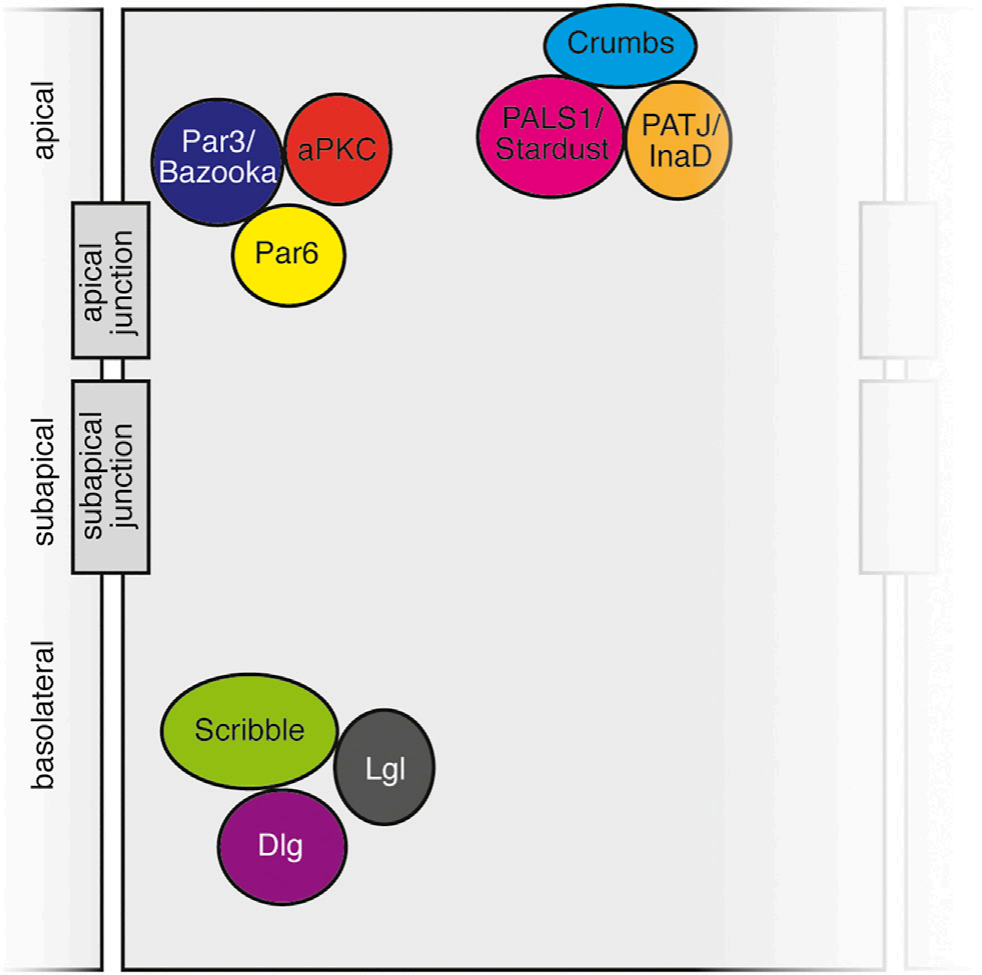
Schematic of polarity complexes in vertebrate and invertebrate cells. Apical Crumbs complex, subapical Par complex, and basolateral Scribble complex depicted with apical junctions (vertebrate tight junctions, invertebrate septate junctions) and subapical junctions (adherens junctions) denoted.

**FIGURE 3:**
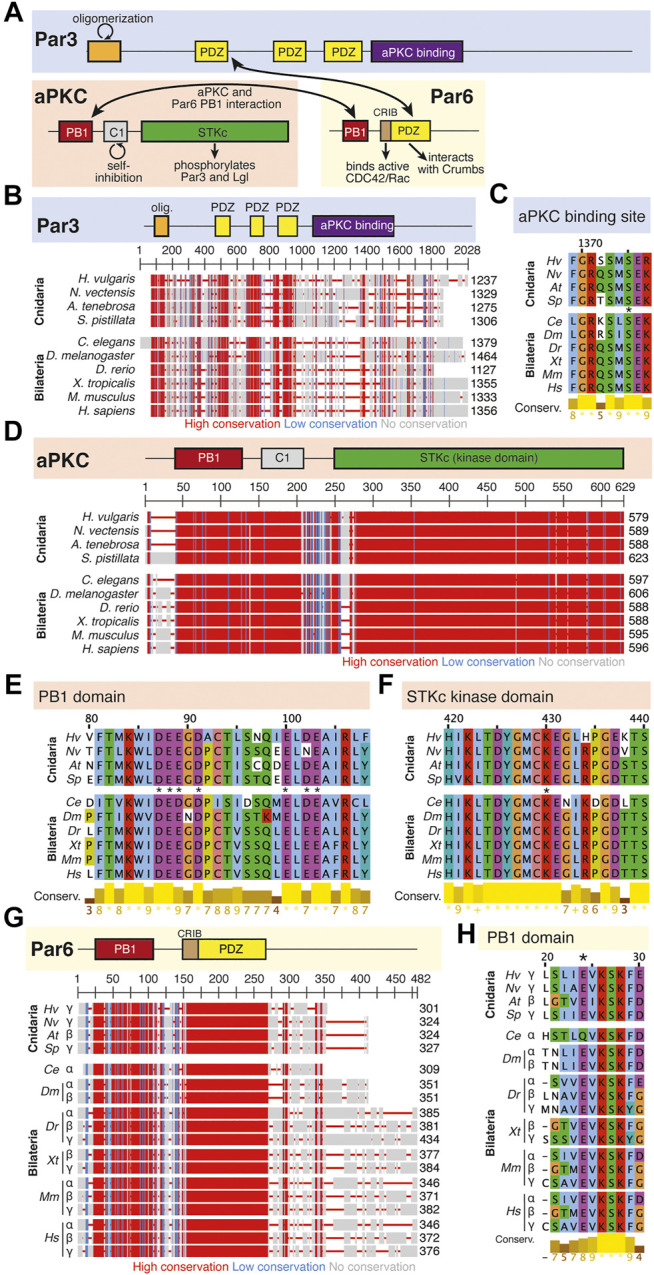
The Par complex defines the subapical domain and is conserved across bilaterians and cnidarians. **(A)** Representation of the human Par complex with Par3, Par6, and aPKC pictured. Functional domains and examples of protein-protein interactions denoted. **(B)** Full protein alignment of Par3 with functional domains depicted in their respective positions. **(C)** Amino acid alignment of the aPKC binding region of Par3. **(D)** Full protein alignment of aPKC with functional domains depicted in their respective positions. **(E-F)** Amino acid alignment of aPKC PB1 domain (E) and STKc kinase domain (F). Critical residues marked with asterisk. **(G)** Full protein alignment of Par6 with functional domains depicted in their respective positions. **(H)** Amino acid alignment of Par6 PB1 domain. Critical residues marked with asterisk. **For all alignments:** COBALT used for all protein alignments. High (red), low (blue), and no conservation (gray) regions denoted. FASTA sequences from COBALT visualized using JalView for amino acid alignments. Hydrophobic (blue), positively charged (red), negatively charged (magenta), aromatic (cyan), and polar (green) amino acids denoted by color, as well as cysteines (pink), glycines (orange), prolines (yellow). Conservation denoted on bottom of alignment. Cnidarians: *Hydra vulgaris* (*Hv*), *Nematostella vectensis* (*Nv*), *Actinia tenebrosa* (*At*), *Stylophora pistillata* (*Sp*). Bilaterians: *Caenorhabditis elegans* (*Ce*), *Drosophila melanogaster* (*Dm*), *Danio rerio* (*Dr*), *Xenopus tropicalis* (*Xt*), *Mus musculus* (*Mm*), *Homo sapiens* (*Hs*). Refer to Tables 1–3 for information regarding sequences used in this figure.

**FIGURE 4:**
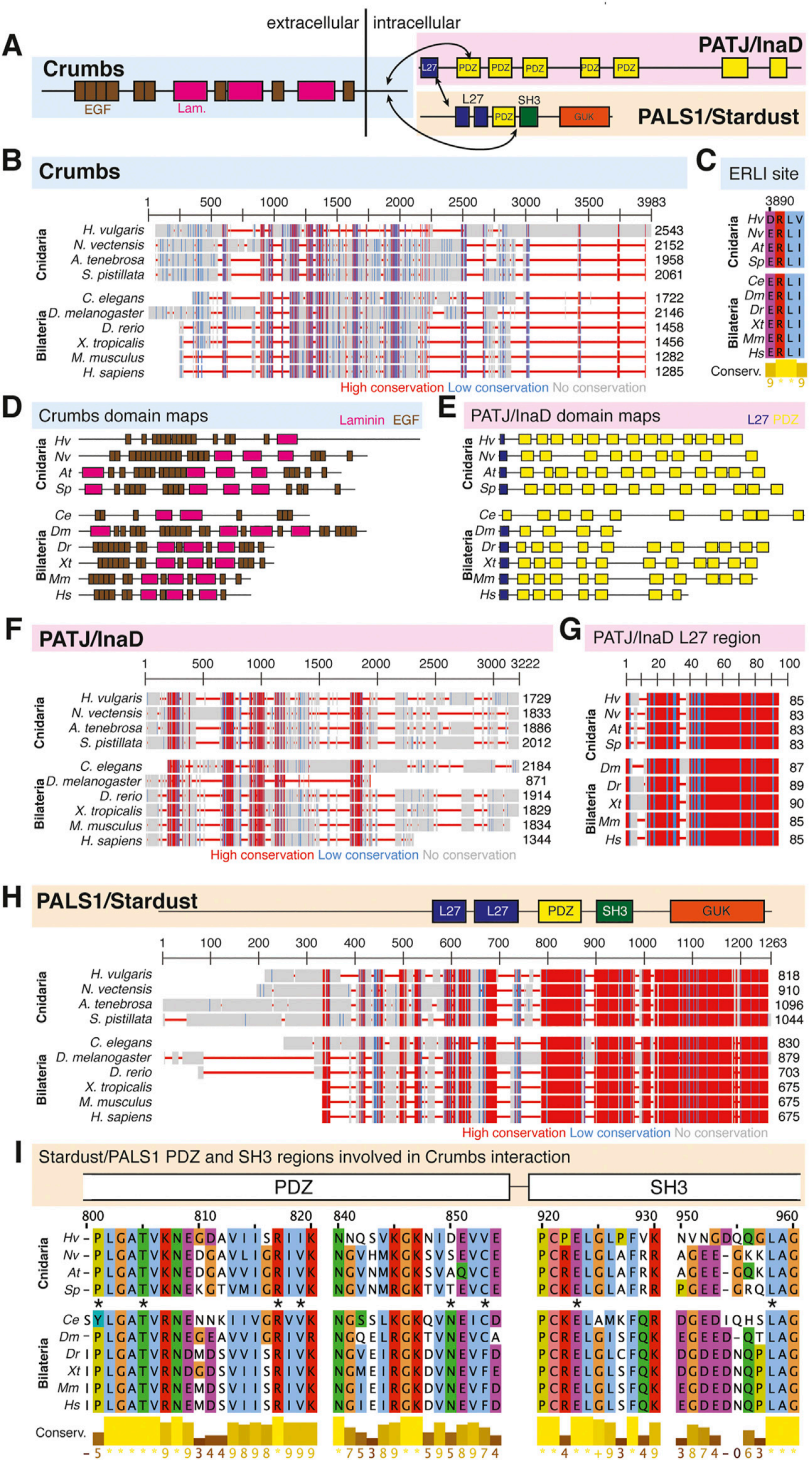
The Crumbs complex defines the apical domain and is conserved across bilaterians and cnidarians. **(A)** Representation of the human Crumbs complex with Crumbs, PALS1/Stardust, PATJ/InaD pictured. Functional domains and examples of protein-protein interactions denoted. **(B)** Full protein alignment of Crumbs. **(C)** Amino acid alignment of the ERLI motif at the Crumbs C-terminus. **(D-E)** Domains maps of Crumbs (D) and PATJ/InaD (E) depicting laminin (magenta, D), EGF (brown, D), L27 (navy, E), and PDZ (yellow, E) domain positions. Protein lengths drawn to scale. **(F)** Full protein alignment of PATJ/InaD. **(G)** Protein alignment of PATJ/InaD L27 domain magnified from (F). **(H)** Full protein alignment of PALS1/Stardust with functional domains depicted in their respective positions. **(I)** Amino acid alignment of regions within PALS1/Stardust PDZ and SH3 domains. Critical residues involved in binding Crumbs marked with asterisk. **For all alignments:** COBALT used for all protein alignments. High (red), low (blue), and no conservation (gray) regions denoted. FASTA sequences from COBALT visualized using JalView for amino acid alignments. Hydrophobic (blue), positively charged (red), negatively charged (magenta), aromatic (cyan), and polar (green) amino acids denoted by color, as well as cysteines (pink), glycines (orange), prolines (yellow). Conservation denoted on bottom of alignment. Cnidarians: *Hydra vulgaris* (*Hv*), *Nematostella vectensis* (*Nv*), *Actinia tenebrosa* (*At*), *Stylophora pistillata* (*Sp*). Bilaterians: *Caenorhabditis elegans* (*Ce*), *Drosophila melanogaster* (*Dm*), *Danio rerio* (*Dr*), *Xenopus tropicalis* (*Xt*), *Mus musculus* (*Mm*), *Homo sapiens* (*Hs*). Refer to Tables 4–6 for information regarding sequences used in this figure.

Lastly, cnidarians such as *Hydra vulgaris* (Chapman et al., 2010; Siebert et al., 2019), the anemone *Nematostella vectensis* (Putnam et al., 2007; Sebe-Pedros et al., 2018), and jellyfish *Clytia hemisphaerica* (Leclere et al., 2019) have been genetically sequenced and/or transcriptionally characterized. This important factor increases the number of genetic tools available for use in these organisms such as CRISPR knockout technology (Ikmi et al., 2014; Lommel et al., 2017; Momose et al., 2018). Components of the Par, Crumbs, and Scribble polarity complexes have been identified and characterized in cnidarian organisms (Figures 1–4), and their high level of conservation with bilaterian systems suggests that studies in cnidarian model systems could contribute to the field of polarity establishment.

**TABLE 1:** aPKC.

Taxa	Organism	NCBI ref seq ID	name
Cnidaria	*Hydra vulgaris*	XP_012559790.1	Predicted: protein kinase C iota type-like
	*Nematostella vectensis*	XP_032242981.1	protein kinase C iota type
	*Actinia tenebrosa*	XP_031568621.1	protein kinase C iota type-like
	*Stylophora pistillata*	XP_022789559.1	protein kinase C iota type-like isoform X3
Bilatera	*Caenorhabditis elegans*	NP_495011.1	Protein kinase C-like 3
	*Drosophila melanogaster*	NP_001036541.1	atypical protein kinase C, isoform C
	*Danio rerio*	NP_571930.2	protein kinase C iota type
	*Xenopus tropicalis*	NP_001012707.1	protein kinase C iota type
	*Mus musculus*	NP_032883.2	protein kinase C iota type
	*Homo sapiens*	NP_002731.4	protein kinase C iota type

**TABLE 2:** Par3.

Taxa	Organism	NCBI ref seq ID	Name
Cnidaria	*Hydra vulgaris*	XP_012559005.1	PREDICTED: uncharacterized protein LOC100212317 isoform X2
	*Nematostella vectensis*	XP_001637950.2	partitioning defective 3 homolog isoform X2
	*Actinia tenebrosa*	XP_031549913.1	partitioning defective 3 homolog isoform X1
	*Stylophora pistillata*	XP_022805323.1	partitioning defective 3 homolog isoform X1
Bilatera	*Caenorhabditis elegans*	NP_001022607.1	Partitioning defective protein 3
	*Drosophila melanogaster*	NP_001334669.1	bazooka, isoform A
	*Danio rerio*	NP_991298.1	par-3 family cell polarity regulator alpha, b
	*Xenopus tropicalis*	XP_004915521.1	partitioning defective 3 homolog isoform X2
	*Mus musculus*	NP_296369.2	partitioning defective 3 homolog isoform 3
	*Homo sapiens*	NP_062565.2	partitioning defective 3 homolog isoform 1

**TABLE 3:** Par6.

Taxa	Organism	NCBI ref seq ID	Name
Cnidaria	*Hydra vulgaris*		Predicted: partitioning defective 6 homolog gamma-like
	*Nematostella vectensis*	XP_032231060.1	partitioning defective 6 homolog gamma
	*Actinia tenebrosa*	XP_031569341.1	partitioning defective 6 homolog beta-like
	*Stylophora pistillata*	XP_022786713.1	partitioning defective 6 homolog gamma-like
Bilatera	*Caenorhabditis elegans*	NP_001040687.1	Partitioning defective protein 6
	*Drosophila melanogaster*	NP_573238.1	par-6, isoform A
		>NP_728094.1	par-6, isoform B
	*Danio rerio*	NP_001093521.2	partitioning defective 6 homolog alpha
		NP_001096145.1	partitioning defective 6 homolog beta
		NP_997728.1	par-6 family cell polarity regulator gamma b
	*Xenopus tropicalis*	NP_001122111.1	partitioning defective 6 homolog beta
		NP_001017338.1	partitioning defective 6 homolog gamma
	*Mus musculus*	NP_062669.2	partitioning defective 6 homolog alpha isoform 1
		NP_067384.2	partitioning defective 6 homolog beta
		NP_444347.3	partitioning defective 6 homolog gamma
	*Homo sapiens*	NP_058644.1	partitioning defective 6 homolog alpha isoform 1
		NP_115910.1	partitioning defective 6 homolog beta
		NP_115899.1	partitioning defective 6 homolog gamma

**TABLE 4:** Crumbs.

Taxa	Organism	NCBI ref seq ID	Name
Cnidaria	*Hydra vulgaris*	XP_012557050.1	Predicted: uncharacterized protein LOC100203132
	*Nematostella vectensis*	XP_032230278.1	protein crumbs isoform X2
	*Actinia tenebrosa*	XP_031551871.1	protein crumbs homolog 1-like
	*Stylophora pistillata*	XP_022801826.1	protein crumbs-like
Bilatera	*Caenorhabditis elegans*	NP_510822.1	CCD66913.1 *Drosophila* CRumBs homolog
	*Drosophila melanogaster*	NP_524480.2	crumbs, isoform A
	*Danio rerio*	NP_001038627.1	protein crumbs homolog 2b precursor
	*Xenopus tropicalis*	XP_002937280.2	protein crumbs homolog 2 isoform X1
	*Mus musculus*	NP_001157038.1	protein crumbs homolog 2 precursor
	*Homo sapiens*	NP_775960.4	protein crumbs homolog 2 precursor

**TABLE 5:** PATJ/InaD.

Taxa	Organism	NCBI ref seq ID	Name
Cnidaria	*Hydra vulgaris*	XP_012558951.1	Predicted: multiple PDZ domain protein-like
	*Nematostella vectensis*	EDO33706.1	predicted protein
	*Actinia tenebrosa*	XP_031574464.1	multiple PDZ domain protein-like
	*Stylophora pistillata*	XP_022779809.1	multiple PDZ domain protein-like isoform X2
Bilatera	*Caenorhabditis elegans*	ABH03415.1	MPZ-1
	*Drosophila melanogaster*	NP_477342.1	patj, isoform C
	*Danio rerio*	XP_009294504.1	inaD-like protein isoform X1
	*Xenopus tropicalis*	XP_002931635.3	inaD-like protein isoform X1
	*Mus musculus*	NP_766284.2	inaD-like protein isoform 1
	*Homo sapiens*	XP_011538771.1	inaD-like protein isoform X12

**TABLE 6:** Stardust/PALS1/MPP5.

Taxa	Organism	NCBI ref seq ID	Name
Cnidaria	*Hydra vulgaris*	XP_012557503.1	Predicted: MAGUK p55 subfamily member 5-like
	*Nematostella vectensis*	XP_032229010.1	MAGUK p55 subfamily member 5 isoform X1
	*Actinia tenebrosa*	XP_031565974.1	uncharacterized protein LOC116301110
	*Stylophora pistillata*	XP_022792787.1	MAGUK p55 subfamily member 5-like
Bilatera	*Caenorhabditis elegans*	NP_001355433.1	MAGUK family
	*Drosophila melanogaster*	NP_001245575.1	stardust, isoform K
	*Danio rerio*	XP_009291449.1	MAGUK p55 subfamily member 5-A isoform X1
	*Xenopus tropicalis*	XP_002937246.1	MAGUK p55 subfamily member 5
	*Mus musculus*	NP_062525.1	protein PALS1
	*Homo sapiens*	NP_071919.2	protein PALS1 isoform 1

Beyond technical advantages, choosing to utilize a cnidarian or other basal model organism provides a simplified setting to investigate research questions within the context of a whole, functional animal. For example, *Hydra vulgaris* features a body plan with two main tissue layers, the ectoderm and endoderm, separated by the mesoglea and interstitial cells. This provides a setting to study processes such as tissue morphogenesis (Hicklin and Wolpert 1973; Maroudas-Sacks et al., 2021), cell differentiation and lineage tracing (Plickert and Kroiher 1988; Takashima, Gold, and Hartenstein 2013; Siebert et al., 2019), and asexual budding mechanisms (Clarkson and Wolpert 1967; Webster and Hamilton 1972) in a simplified, yet multi-tissue, context. Within the wide spectrum of biological research models to choose from, cnidarians and other basal animals represent an important niche between *ex vivo* cultured systems and more complicated *in vivo* bilaterian model organisms such as mouse, zebrafish, or *Drosophila*.

Cnidarians occupy an advantageous position within the evolutionary tree in a group historically referred to as Epitheliozoa. This subset of organisms is comprised of bilaterians, cnidarians, ctenophores, and placozoans, all of which are considered animals that have “true tissues” (Ax 1995). The exact definition of an epitheliozoan seems to be controversial in the literature. Some sources cite the presence of belt desmosomes as a requirement for this grouping (Ax 1995; Dohrmann and Worheide 2013), while others define “true tissues” as those that are connected by tight junctions (septate junctions in invertebrates) (Ganot et al., 2015). It is also debated whether they have the appropriate proteins or the ability to properly build these junctional complexes (Sperling, Peterson, and Pisani 2009). For example, sponges can be excluded since they do not have proper belt desmosomes (Leys and Riesgo 2012). Additionally, poriferans only have one tissue type within their body, raising the argument that they cannot have true epithelial tissues if they are not creating barriers between different tissue types. While the poriferan group is controversial when it comes to its relationship to epitheliozoans, cnidarians have been placed in this clade through a variety of genomic and phenotypic analyses (Ax 1995; Zrzavy et al., 1998; Sperling, Peterson, and Pisani 2009; Dohrmann and Worheide 2013; Ganot et al., 2015). Epithelia are therefore an ancient characteristic and important enough in biological evolution that a phylogenetic grouping has been created for organisms with epithelial tissues. Within this group, cnidarians are a more basal group of organisms, making them an option to study epithelial polarity establishment and maintenance in a simple epitheliozoan.

### Polarity and Epithelialization Studies in Cnidarian Models

Mechanisms controlling planar cell polarity are conserved in cnidarians. Briefly, the core planar cell polarity pathway involves the asymmetric distribution of several critical proteins to distinguish one side of the cell from the other along the larger-scale axis of the tissue and embryo. Some of these proteins include Frizzled, Strabismus/Van Gogh, Flamingo, Dishevelled, Prickle, and Diego (Devenport 2014). These evolutionarily conserved proteins are required for proper morphogenesis and development in several cnidarian species. For example, Strabismus, Frizzled, and Dishevelled are required for *Nematostella vectensis* invagination (Kumburegama, Wijesena, and Wikramanayake 2008; Kumburegama et al., 2011; Wijesena and Martindale 2018; Technau 2020; Wijesena et al., 2022), ciliated epithelium development in *Clytia hemispherica* (Momose and Houliston 2007; Momose, Derelle, and Houliston 2008; Momose, Kraus, and Houliston 2012; Lapebie et al., 2014), and tissue evagination dependent on Strabismus, Dishevelled, and Frizzled in *Hydra vulgaris* (Philipp et al., 2009). Additionally, the Fat-Dachsous polarity pathway utilizes an asymmetry in the localization of the protocadherins Fat and Dachsous to create cellular and tissue polarity (Devenport 2014), and these components have been similarly identified and studied in *Hydra* and *Nematostella* (Magie and Martindale 2008; Hulpiau and van Roy 2011; Tucker and Adams 2014; Gul et al., 2017). While fewer studies concern the mechanisms driving apicobasal polarity cnidarians, the conservation of PCP factors suggests that apicobasal polarity complexes and mechanisms are also likely to be conserved in cnidarians.

***Hydra vulgaris*** *Hydra* are the oldest cnidarian model system used in research (Figure 1A), first studied by Abraham Trembley in 1744 (Trembley, 1744). The *Hydra* body column is largely comprised of two epithelial cell layers, the ectoderm and endoderm, with various types of interstitial cells positioned between. Additional cell types are interspersed between the epithelial cells, including gland cells in endoderm, nematocytes and nematoblasts in the ectoderm, and various neural cell types found in both layers (Technau and Steele 2011). The mesoglea is positioned between the two layers, composed of a thick extracellular matrix secreted by the endoderm and ectoderm (Epp, Smid, and Tardent 1986; Sarras et al., 1993). Basal myofibrils present in both cell layers run in orthogonal directions, with those in the endoderm running circumferentially around the body column, and those of the ectoderm running longitudinally along the body axis (Otto 1977). The endoderm is sometimes referred to as the gastrodermis due to the presence of gland cells that aid in digestion. *Hydra* is even referred to as a “living gut” due to their simple digestive system that spans the body column, and therefore much of the *Hydra* body plan in general (Vogg, Galliot, and Tsiairis 2019). *Hydra* feature a robust asexual reproduction process called budding, which has been used to study developmental programming and head determination. Epithelialization is an important process in both budding and regeneration, which is one of the main reasons that *Hydra* is a promising model for the study of polarity establishment and cell sorting (Seybold, Salvenmoser, and Hobmayer 2016).

One of the main advantages of the *Hydra* model system is their ability to reassemble after dissociation and subsequent reaggregation. This reaggregation can occur with minimal cell numbers, as only approximately 5–15 cells are necessary to create a head organizer within the population, allowing for a full *Hydra* to grow out of the small cell cluster (Technau et al., 2000). The patterning mechanism that defines the head, foot, and tentacle regions of the *Hydra* have been modelled using simulations such as the Meinhardt reaction-diffusion model, where these regions positioned with respect to one another by gradients of inhibitory or activating signals that determine the *Hydra* body axis (MacWilliams 1982; Meinhardt 1993).

*Hydra* reaggregation experiments have been used to understand how cells sort within a mixed population. Such studies suggest that factors such as the capacity for epithelialization (Skokan, Vale, and McKinley 2020), cell surface tension (Cochet-Escartin et al., 2017), interfacial tension and cell-cell adhesiveness (Technau and Holstein 1992) dictate how cells will sort into layers within an aggregate. Ectodermal engulfment of the endoderm in *Hydra* is compared to the morphogenetic process of epiboly in other organisms, where epithelial tissue undergoes a spreading process in the early development of vertebrates such as fish and amphibians (Piccolo 2013). Similarly, the *Hydra* ectodermal cells engulfed the endodermal cell cluster when they came into contact, reforming the bilayer epithelium that is typically found in *Hydra* (Kishimoto, Murate, and Sugiyama 1996). As shown in these examples, the ability to completely dissociate *Hydra* tissues provides a setting to study the physical and biochemical characteristics of these cells to determine how they will behave when combined in a tissue with other cell populations, and how that multicellular tissue behaves as a whole during development.

Epithelialization as it relates to cell-cell adhesion establishment has also been extensively studied using *Hydra* reaggregation experiments. The process of cell-cell adhesion reestablishment was documented through electron microscopy to determine the order in which adhesion complexes are created. In this context, the apical-basal axis of developing epithelial cells elongates, followed by septate junction, gap junction, mesoglea, and hemidesmosome-like junction development. Interestingly, the process of planar cell polarity establishment begins before apical-basal polarity establishment has completed (Seybold, Salvenmoser, and Hobmayer 2016). Apicobasal polarity establishment both influences and is influenced by the setup of cell-cell adhesions, and these molecular players have been identified and studied in *Hydra* (Buzgariu et al., 2015).

***Nematostella vectensis*** The sea anemone *Nematostella vectensis* is one of the more commonly utilized cnidarian model systems outside of hydroids (Figure 1B). *Nematostella* was the first cnidarian to be genetically sequenced and while it has a relatively small genome, there are remarkable similarities to the genomes of humans and other bilaterian vertebrates (Putnam et al., 2007). For example, almost half of 27,000 predicted protein coding transcripts have clear orthologs to protostomes, deuterostomes, or both, and the number of exons and splice sites are nearly identical to humans (Tucker and Adams 2014). Interestingly, a comprehensive single-cell analysis of whole *Nematostella* animals found that many genetic similarities are shared between cnidarians and vertebrate bilaterians that are not present in invertebrate bilaterians, which include common model systems such as *C. elegans* and *Drosophila* (Putnam et al., 2007; Sebe-Pedros et al., 2018). For example, DNA methylation is absent in both *Drosophila* and *C. elegans* but occurs in *Nematostella* and other invertebrate organisms (Feng et al., 2010; Zemach et al., 2010; Zemach and Zilberman 2010; Schwaiger et al., 2014).

Synchronous cell divisions increase the cell mass of the embryo during early *Nematostella* development. The localization of Par3/Bazooka and Par6 oscillates in time with these cell divisions, moving to cell surfaces and cell-cell interfaces between divisions and away from this location during divisions (Ragkousi et al., 2017; Doerr and Ragkousi 2019). Par6 localizes to the apical cortex during interphase, and Par3/Bazooka can be found at subapical cell-cell contacts during this time. However, consistent with previous work in *Drosophila* (Bergstralh, Lovegrove, and St Johnston 2013), neither protein is detected at these sites during mitosis (Ragkousi et al., 2017; Doerr and Ragkousi 2019). This points to a mechanism that can quickly dismantle and reestablish epithelial polarity between cell divisions to preserve epithelial integrity during tissue growth. Interestingly, components of the Par system are not present in the endomesodermal epithelial tissue during gastrulation in *Nematostella*, despite being present in blastula cells earlier in development (Salinas-Saavedra et al., 2015; Salinas-Saavedra, Rock, and Martindale 2018; Salinas-Saavedra and Martindale 2020). This coincides with the absence of adherens junctions in this same tissue, suggesting different mechanisms of cell adhesion in these adjacent tissues (Salinas-Saavedra, Rock, and Martindale 2018). This loss of both cell polarity and cell-cell adhesions points to EMT occurring in this specific population of cells during this stage of development (Whiteman et al., 2008; Lim and Thiery 2012). Furthermore, apical polarity proteins such as Par1, Par3/Bazooka, Par6, aPKC, and Lgl only become asymmetrically distributed to their respective membrane domains later on in development, whereas they are localized along the cytoplasm and microtubule cytoskeleton during early developmental stages (Salinas-Saavedra et al., 2015).

Additional studies have determined the role of cadherins during *Nematostella* tissue morphogenesis. The cadherin-catenin complex is conserved in *Nematostella* and is required at the adherens junctions for proper embryo development and later tissue morphogenesis (Clarke et al., 2016; Nathaniel Clarke, Lowe, and James Nelson 2019). For example, Cadherin1 and Cadherin3 are expressed at different times during development, marking the transition from blastoderm to distinct germ layer formation. Disruption of this cadherin expression pattern results in a loss of tissue integrity and improper embryogenesis (Pukhlyakova et al., 2019).

***Clytia hemisphaerica*** The jellyfish *Clytia hemisphaerica* (Figure 1C) is an increasingly popular cnidarian model for the study of tissue development, wound healing, and regeneration (Kamran et al., 2017; Malamy and Shribak 2018; Kraus, Chevalier, and Houliston 2020; Sinigaglia et al., 2020). Its genome has been recently sequenced, and previous studies have also characterized the *Clytia* transcriptome through single cell RNA-sequencing (Leclere et al., 2019; Chari et al., 2021). *Clytia* are frequently used to study wound healing, a component of which is epithelialization. The epithelial layer covering the surface of *Clytia medusa* is composed of a flat, squamous monolayer, allowing for imaging with DIC (differential interference contrast) microscopy (Malamy and Shribak 2018). As a result, the migration of epithelial cells during development, wound healing, and regeneration has been carefully analyzed to determine the physical steps and chemical mechanisms involved (Malamy and Shribak 2018; Kraus, Chevalier, and Houliston 2020; Sinigaglia et al., 2020). These instances of epithelial-to-mesenchymal transitions, as well as the dedifferentiation events that occur in other jellyfish species (Lin, Grigoriev, and Spencer 2000; Estephane and Anctil 2010), make *Clytia* a promising model system for use in the study of both the establishment and breakdown of apical cell polarity. An additional advantage to this system is that *Clytia* components can be cultured outside the body for additional technical assays. For example, the female gonads of *Clytia* have been cultured to determine the mRNA gradients required to set up the body axes of the developing animal (Amiel and Houliston 2009).

Studies using *Clytia* have investigated the intersection between apicobasal polarity establishment and body axis establishment. Rapid synchronous cell divisions during early development increase embryonic cell mass prior to tissue layer specification. After the midblastula stage, cell divisions become asynchronous as cells begin to polarize and adopt a more columnar shape with nuclei that begin to localize towards the apical surface. At this point, ingression and gastrulation begin and the ectoderm and endoderm start to take form. This occurs through a population of cells called bottle cells that undergo EMT (epithelial to mesenchymal transition), change their shape to detach from the epithelial layer into the blastocoel, and adopt a mesenchymal morphology. These bottle cells do not undergo EMT and inward migration at the same time, allowing for many cells at varying stages of this process to be observed and characterized simultaneously. After 24 h post-ingression, these cells have reorganized to create the endodermal epithelial layer inside the developing embryo (Kraus, Chevalier, and Houliston 2020). This creates an opportunity to effectively study the process of polarity establishment in the early *Clytia* embryo, as well as its reverse process in EMT later during gastrulation.

### Apicobasal Polarity Establishment and Maintenance

While the exact mechanism of polarity establishment on a molecular level is not completely understood, there are a few important steps that are consistent across species. Firstly, three cortical polarity complexes (Par, Crumbs, and Scribble) interact to eventually localize properly to their respective domains (Figure 2). This triggers downstream pathways to continue setting up the necessary molecular components at each cellular surface. Secondly, cell-cell adhesions are established to attach cells to one another and provide tissue structural integrity, as well as an avenue for communication and materials transport between cells. This includes tight junctions (known as septate junctions in invertebrates), adherens junctions, and desmosomal junctions. The organization of polarity complexes and creation of cell-cell junctions are interconnected processes, with each process influencing and being influenced by the other (Rodriguez-Boulan and Macara 2014). While this review focuses mainly on the role of the Crumbs, Par, and Scribble complexes, a comprehensive review of cell-cell adhesion molecules has been published by Tepass et al. (2001).

Cell polarity in epithelia is driven by mutual antagonism between cortical factors. Work across multiple systems has demonstrated the importance of at least three conserved protein complexes: the Par (Par6, aPKC, Par3/Bazooka) and Crumbs (Crumbs, Stardust, PATJ) complexes that are found at the apical and subapical surface, respectively, and the Scribble module (Discs Large, Lethal Giant Larvae, Scribble) that is sequestered to the basolateral membrane (Tepass, Theres, and Knust 1990; Knust, Tepass, and Wodarz 1993; Etemad-Moghadam, Guo, and Kemphues 1995; Tabuse et al., 1998; Bilder, Li, and Perrimon 2000; Bilder and Perrimon 2000). While the exact relationships between individual complex members continue to be elucidated, an emerging theme is that polarity is maintained by an exceedingly complex network of mutual antagonism. This also includes proteins outside the canonical Par, Crumbs, and Scribble complexes, such as Yurt (Laprise et al., 2006; Laprise et al., 2009) and Par1 (Benton and St Johnston 2003).

The Par complex includes Par6, Par3/Bazooka, and aPKC (atypical protein kinase C, Figure 3). The spatial relationship between Par proteins is conserved throughout animals, but interactions between the components are complex and historically have been difficult to parse out. In *Drosophila*, Par3/Bazooka, Par6, and aPKC localize apically (Kuchinke, Grawe, and Knust 1998; Wodarz et al., 2000; Petronczki and Knoblich 2001; Morais-de-Sa, Mirouse, and St Johnston 2010). The size of the apical domain is regulated through a negative feedback mechanism between Crumbs and Yurt, a basolateral protein that is recruited to apical membranes towards the end of epithelial development (Laprise et al., 2006; Laprise et al., 2009).

The Crumbs complex has four known components, Crumbs, Stardust/PALS1 (protein associated with Lin7 1), and PATJ/InaD (PALS1-associated tight junction protein/inactivation no afterpotential D, Figure 4) (Bachmann et al., 2001; Bachmann et al. 2004; Bachmann et al. 2008). Crumbs is partially responsible for the establishment of the apical domain in epithelial cells (Wodarz et al., 1995), and all components of the Crumbs complex are thought to be required for tight junction formation in mammals (Tan et al., 2020).

**FIGURE 5:**
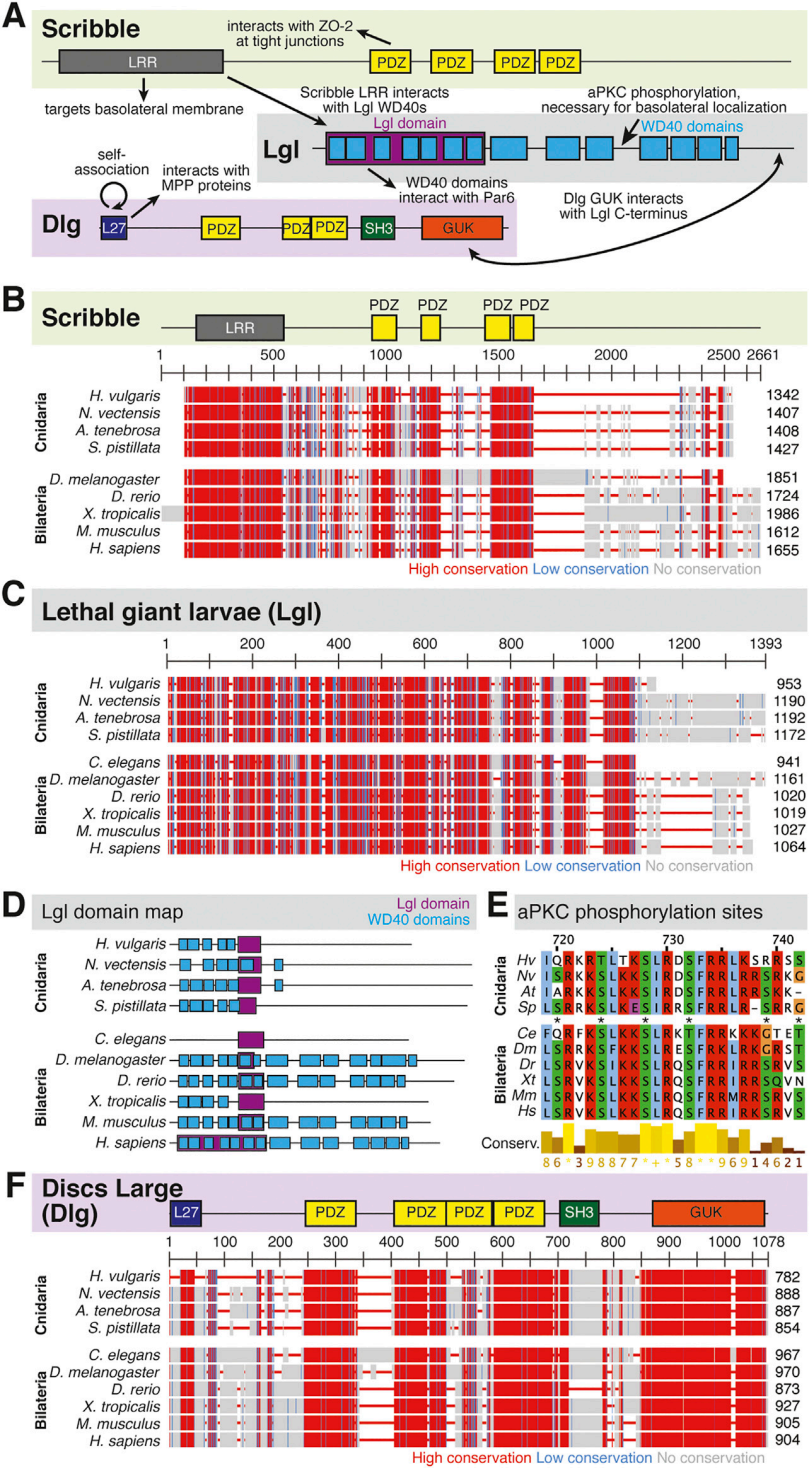
The Scribble complex defines the basolateral domain and is conserved across bilaterians and cnidarians. **(A)** Representation of the human Scribble complex with Scribble, Discs Large (Dlg), and Lethal Giant Larvae (Lgl) pictured. Functional domains and examples of protein-protein interactions denoted. **(B,C)** Full protein alignments of Scribble (B) and Lethal Giant Larvae (Lgl, C). Scribble functional domains depicted in their respective positions within alignment. **(D)** Lgl domains maps depicting WD40 (cyan) and Lgl (violet) positions. Protein lengths drawn to scale. **(E)** Amino acid alignment of Lgl region phosphorylated by aPKC. Critical residues depicted with asterisks. **(F)** Full protein alignment of Discs Large (Dlg). functional domains depicted in their respective positions within alignment. **For all alignments:** COBALT used for all protein alignments. High (red), low (blue), and no conservation (gray) regions denoted. FASTA sequences from COBALT visualized using JalView for amino acid alignments. Hydrophobic (blue), positively charged (red), negatively charged (magenta), aromatic (cyan), and polar (green) amino acids denoted by color, as well as cysteines (pink), glycines (orange), prolines (yellow). Conservation denoted on bottom of alignment. Cnidarians: *Hydra vulgaris* (*Hv*), *Nematostella vectensis* (*Nv*), *Actinia tenebrosa* (*At*), *Stylophora pistillata* (*Sp*). Bilaterians: *Caenorhabditis elegans* (*Ce*), *Drosophila melanogaster* (*Dm*), *Danio rerio* (*Dr*), *Xenopus tropicalis* (*Xt*), *Mus musculus* (*Mm*), *Homo sapiens* (*Hs*). Refer to Tables 7–9 for information regarding sequences used in this figure.

Scribble module factors localize to the basolateral membrane (Figure 5) (Bilder and Perrimon 2000; St Johnston and Ahringer 2010). In the *Drosophila* follicular epithelium, Discs Large (Dlg) localizes Scribble to the cortex via the Dlg SH3 domain. Lethal Giant Larvae (Lgl) is a known inhibitor of Par complex component aPKC, and this inhibition can occur here through the interaction of Lgl with Dlg and Scribble. This mechanism then confines the Scribble complex components to the basolateral domain, and aPKC along with other Par complex components to the apical domain above (Khoury and Bilder 2020; Ventura et al., 2020). Lgl is involved in mutual antagonism with aPKC, which phosphorylates Lgl to exclude it from the apical cortex. Conversely, Lgl inhibits aPKC from the lateral domain (Lee, Robinson, and Doe 2006; Atwood and Prehoda 2009; Ventura et al., 2020).

**TABLE 7:** Discs Large (Dlg).

Taxa	Organism	NCBI ref seq ID	Name
Cnidaria	*Hydra vulgaris*	XP_012556528.1	PREDICTED: disks large homolog 1-like
	*Nematostella vectensis*	XP_001638123.2	disks large homolog 1
	*Actinia tenebrosa*	XP_031552782.1	disks large homolog 1-like isoform X2
	*Stylophora pistillata*	XP_022791231.1	disks large homolog 1-like isoform X2
Bilatera	*Caenorhabditis elegans*	NP_001024431.1	Disks large homolog 1
	*Drosophila melanogaster*	NP_996406.1	discs large 1, isoform B
	*Danio rerio*	NP_955820.1	disks large homolog 1
	*Xenopus tropicalis*	NP_001039116.1	disks large homolog 1
	*Mus musculus*	NP_001239364.1	disks large homolog 1 isoform 4
	*Homo sapiens*	NP_001091894.1	disks large homolog 1 isoform 1

**TABLE 8:** Lethal Giant Larvae (Lgl).

Taxa	Organism	NCBI ref seq ID	Name
Cnidaria	*Hydra vulgaris*	XP_012555599.1	PREDICTED: lethal (2) giant larvae protein homolog 1 isoform X1
	*Nematostella vectensis*	XP_032221124.1	lethal (2) giant larvae protein homolog 1 isoform X1
	*Actinia tenebrosa*	XP_031560162.1	lethal (2) giant larvae protein homolog 1-like isoform X1
	*Stylophora pistillata*	XP_022802656.1	lethal (2) giant larvae protein homolog 2-like
Bilatera	*Caenorhabditis elegans*	NP_508169.2	LLGL domain-containing protein
	*Drosophila melanogaster*	NP_001245801.1	lethal (2) giant larvae, isoform G
	*Danio rerio*	NP_997747.1	LLGL scribble cell polarity complex component
	*Xenopus tropicalis*	XP_012827183.1	LLGL scribble cell polarity complex component 2 isoform X1
	*Mus musculus*	NP_663413.2	LLGL scribble cell polarity complex component 2 isoform 1
	*Homo sapiens*	NP_004131.4	lethal (2) giant larvae protein homolog 1

**TABLE 9:** Scribble.

Taxa	Organism	NCBI ref seq ID	Name
Cnidaria	*Hydra vulgaris*	XP_004209241.2	Predicted: protein scribble homolog isoform X1
	*Nematostella vectensis*	XP_032228868.1	protein scribble homolog isoform X1
	*Actinia tenebrosa*	XP_031556444.1	protein scribble homolog
	*Stylophora pistillata*	XP_022783798.1	protein scribble homolog isoform X1
Bilatera	*Caenorhabditis elegans*	CAB91651.1	LET-413 protein
	*Drosophila melanogaster*	NP_524754.2	scribble, isoform D
	*Danio rerio*	NP_001007176.1	protein scribble homolog
	*Xenopus tropicalis*	XP_031759452.1	protein scribble homolog isoform X1
	*Mus musculus*	NP_001297472.1	protein scribble homolog isoform 3
	*Homo sapiens*	NP_874365.3	protein scribble homolog isoform a

Cell-cell adhesion molecules also play an important role in polarity establishment and maintenance in cooperation with the three polarity complexes mentioned above. For example, adherens junctions can drive apical polarity establishment (Nejsum and Nelson 2007; Desai, Harmon, and Green 2009) in cells, but cell polarity can also govern adherens junction formation in other settings (Qin et al., 2005). While this complicated relationship is still being investigated, it is clear that both cell-cell adhesion and cell polarity establishment are interconnected processes during epithelial tissue morphogenesis and development (Coopman and Djiane 2016).

Most information in the field of polarity establishment and maintenance have come from studies in complex animal systems like *Drosophila* and *C. elegans*, or in cultured systems such as MDCK cells (Etemad-Moghadam, Guo, and Kemphues 1995; Wodarz et al., 1995; Kuchinke, Grawe, and Knust 1998; Tabuse et al., 1998; Wodarz et al., 2000; Petronczki and Knoblich 2001; Martin-Belmonte et al., 2007; Bryant et al., 2010; Morais-de-Sa, Mirouse, and St Johnston 2010; Bergstralh, Lovegrove, and St Johnston 2013; Khoury and Bilder 2020; Ventura et al., 2020). While these studies have contributed a great amount of information to our understanding of epithelial polarization, the addition of more diverse model systems to this body of work would continue to push this field forward. Therefore, it is advantageous to consider cnidarian models and others outside the bilaterian group for future studies.

### Identification and Function of Polarity Regulators in Cnidarians

To demonstrate the utility of cnidarians as a model system for the study of polarity, we and others have undertaken phylogenetic analysis to test whether polarity regulators are conserved. Components of the Par, Crumbs, and Scribble complex have been identified in many organisms outside the bilaterian clade, including several cnidarian species such as *Hydra vulgaris* and *Nematostella vectensis* (Ragkousi et al., 2017; Doerr and Ragkousi 2019; Schiller and Bergstralh 2021)*.* Several important functional domains within these three polarity complexes are conserved between bilaterian and cnidarian organisms, making cnidarian organisms promising models for the study of polarity establishment and maintenance.

Within the Par Complex, aPKC, Par6, and Par3/Bazooka are highly conserved across several bilaterian and cnidarian species (Figure 3). Par3/Bazooka features a specific serine residue within the aPKC binding region that is the site of aPKC phosphorylation, which is vital for the function of the whole Par complex (Soriano et al., 2016; Nagai-Tamai et al., 2002). Although the rest of the protein alignment denotes increased variability, this specific serine residue is present in all organisms tested (Figure 3B, C, S1375). High levels of conservation were found across the alignment of aPKC (Figure 3D). Within aPKC, several specific residues are conserved that are required for the function of the PB1 domain, which interacts with Par6 (Figure 3E). Additionally, a specific lysine residue is required for the kinase function of the STKc (serine/threonine protein kinase catalytic) domain (Li et al., 1995). In addition to high levels of conservation across the whole domain (Figure 3D), this specific invariable lysine residue is conserved across the ten organisms investigated (Figure 3F). Par6 is also highly conserved across both the ten species investigated as well as multiple Par6 isoforms (Figure 3G), including the PB1 region required for interaction with aPKC (Figure 3H). This suggests that the molecular mechanisms driving Par complex function during polarity establishment are conserved across bilaterian and cnidarian organisms.

Overall, components of the Crumbs complex appear to be conserved between the bilaterian and cnidarian species investigated (Figure 4A). Although a protein alignment of Crumbs seems to suggest a low level of sequence conservation (Figure 4B), further examination revealed that the vital EGF and laminin G-like domains are present in all ten species, but in different locations and numbers (Figure 4D). These extracellular domains facilitate protein-protein interactions within the Crumbs complex (Tepass, Theres, and Knust 1990; Bulgakova and Knust 2009; Thompson, Pichaud, and Roper 2013; Rothberg et al., 1988; den Hollander et al., 1999; Sasaki et al., 1988; Omori and Malicki 2006). Additionally, the ERLI motif located at the C-terminus of the Crumbs protein is highly conserved across both cnidarians and bilaterians. This sequence allows for the interaction of Crumbs with the other components of the Crumbs complex, Stardust/PALS1 and PATJ/InaD. Similarly, PATJ/InaD features a highly conserved L27 domain, but the number of subsequent PDZ domains differs from species to species (Figures 4E–G). Lastly, Stardust/PALS1 demonstrates high levels of protein conservation, especially in the region of the PDZ and SH3 domains that facilitate its interaction with Crumbs (Figures 4H, I). These results show that although there is variability between Crumbs isoforms in various species, the functional domains are largely conserved. Therefore, it is likely that Crumbs function in apical polarity establishment and maintenance is conserved as well.

Components of the Scribble complex are highly conserved across bilaterian and cnidarian organisms (Schiller and Bergstralh 2021) (Figure 5A). Both Scribble and Dlg contain multiple PDZ domains with high levels of conservation. These PDZ domains are vital to protein-protein interactions that include Scribble and Dlg within the polarity establishment pathway and others (Kallay et al., 2006; Takizawa et al., 2006; How et al., 2019; Bilder 2003; Bilder, Li, and Perrimon 2000; Bilder, Schober, and Perrimon 2003; Sotelo et al., 2012; Matsumine et al., 1996; Makino et al., 1997; Subbaiah et al., 2012). Lgl displays varying positions of its LGL domain, as well as different numbers of WD40 domains between species (Figures 5C,D). Despite these differences, the aPKC phosphorylation site towards the middle of the protein is highly conserved (Figure 5E). Additionally, the domains of Dlg are highly conserved including the vital GUK domain, which facilitates interaction between Dlg and phosphorylated Pins/LGN/GPSM2 among other possible functions (Johnston et al., 2009; Johnston et al., 2011; Anderson et al., 2016; Schiller and Bergstralh 2021) (Figure 5F). This suggests that both the polarity and spindle orientation mechanisms of Dlg are evolutionarily conserved, as well as the overall function of the Scribble complex.

## Conclusion

Despite the extensive number of epithelialization studies in the past several decades, the information obtained has come from a limited pool of model systems. While bilaterian systems have been incredibly useful in identifying the first polarity proteins and their respective pathways, the complexity of these organisms has mostly limited these studies to early embryogenesis. Alternatively, cultured settings have given a simplified model in which to test the role of these polarity factors during epithelialization, however these settings are not physiologically representative of what may occur in a whole, living organism. Therefore, it would be advantageous to look outside the bilaterian clade for candidate model systems in which to continue and supplement these existing studies. This review has highlighted the technical, genetic, and evolutionary evidence that supports the use of cnidarian model organisms in future studies of cell polarity establishment and maintenance.
